# Does cognitive capital reduce the risk of cognitive decline in later life?

**DOI:** 10.1093/geroni/igaf115

**Published:** 2025-10-15

**Authors:** Kenneth F Ferraro, Bing Han

**Affiliations:** Department of Sociology, Purdue University, West Lafayette, Indiana, United States; Center on Aging and the Life Course, Purdue University, West Lafayette, Indiana, United States; Department of Sociology, Purdue University, West Lafayette, Indiana, United States; Center on Aging and the Life Course, Purdue University, West Lafayette, Indiana, United States

**Keywords:** Cognitive resources, Neighborhood context, Cognitive function, Dementia risk

## Abstract

**Background and Objectives:**

Although considerable evidence shows that various neighborhood characteristics are related to cognitive function, we propose the concept of cognitive capital as a theoretically informed and parsimonious way to guide research on how neighborhood contextual factors may influence cognitive function in later life.

**Research Design and Methods:**

Data in 2010 from the National Neighborhood Data Archive were linked to data from the Health and Retirement Study (2010–2018). Cognitive function was measured with a modified version of the Telephone Interview for Cognitive Status. Using a latent variable modeling approach, cognitive capital was measured with eight indicators of neighborhood context based on census tracts (e.g., museums, fitness centers).

**Results:**

Trajectory analyses revealed that adults between ages 60 and 82 residing in areas with greater cognitive capital manifested higher baseline cognitive function and later onset of cognitive decline compared to those with low cognitive capital.

**Discussion and Implications:**

Cognitive capital is a useful conceptual framework for (a) studying the relationship between neighborhood characteristics and trajectories of cognitive function and (b) designing effective interventions to preserve cognitive function during later life.

Innovation and Translational Significance: This study develops the concept of cognitive capital and examines whether it helps older people maintain higher levels of cognitive function. We find that cognitive capital in one’s neighborhood is prospectively associated with cognitive function among adults ages 60 through 82. Interventions based on the potential influence of cognitive capital should account for the “60-80” window of opportunity reported herein. Beyond that window, additional resources are needed to preserve cognition.

## Background and objectives

The global imprint of dementia is significant, with an estimated 55 million people worldwide living with Alzheimer’s disease or related dementias (Alzheimer’s Association, 2024). In the United States, Alzheimer’s disease has been one of the top 10 causes of death for more than 20 years (Anderson, 2002; Heron, 2017). Awareness of the impact of Alzheimer’s disease has spawned an impressive array of studies to identify biological mechanisms leading to this nervous system disease. Although smaller in number, there is also emergent literature on how neighborhood context influences the risk of cognitive decline during later life.

Most studies over the past decade show that neighborhood factors, such as higher socioeconomic status (SES) and amenities, are associated with lower rates of cognitive impairment (Clarke et al., 2012; Finlay et al., 2021b, 2022; Lee & Waite, 2018; Wu et al., 2017), prompting new research questions and approaches. For instance, all five of these empirical studies used cross-sectional data, although prior systematic reviews on the topic have called for longitudinal analyses to disentangle whether the association arises from neighborhood factors influencing cognition or the reverse—such as when older adults without cognitive impairment move to neighborhoods with higher SES and/or more amenities (Song et al., 2024; Wu et al., 2015). Investigators have also examined a wide range of neighborhood characteristics, including SES, recreation centers, and coffee shops, but questions remain about which of these factors are most important for retaining cognitive function in later life and why.

We seek to contribute to the literature on the topic in two main ways. First, we develop a conceptual framework to identify neighborhood characteristics that might plausibly be related to cognitive function in later life. Second, we build on exemplars of longitudinal research on the topic to prospectively assess changes in cognitive function (e.g., Besser et al., 2021; Choi et al., 2024; Clarke et al., 2015; Finlay et al., 2021a; Wörn et al., 2017). Although many studies have found associations between selected neighborhood characteristics and cognitive function, the use of longitudinal data enables us to pinpoint when—at what ages—these influences are manifest. We begin with consideration of theoretical perspectives on whether neighborhood characteristics reduce the risk of cognitive decline in later life.

### Theoretical framework

A range of neighborhood characteristics has been studied in recent years, with dozens of indicators examined in research on neighborhood context and cognitive function in later life (Song et al., 2024). Some studies focus on a single neighborhood characteristic, such as educational attainment, based on the expectation that living in a neighborhood with a higher proportion of college graduates is beneficial, regardless of one’s own educational level (Wight et al., 2006). However, more recent studies have linked multiple neighborhood indicators, such as educational attainment and proximity to museums, to individual-level survey data (Choi et al., 2024; Clarke et al., 2012; Finlay et al., 2022). Both approaches have been valuable, but studies incorporating multiple neighborhood indicators must address whether to treat each indicator as an individual predictor of cognitive function or to combine them into one or more constructs.

Cognitive capital is a concept that has received limited attention in the research literature but holds significant potential for scholars studying the relationship between multiple neighborhood characteristics and cognitive function in later life. Unfortunately, few scholars explicitly define cognitive capital. Additionally, there is little consensus across empirical studies on how to operationalize the concept. Given this inconsistency, we aim to bring clarity to the term by (a) defining it, (b) articulating key elements for measuring it, and (c) assessing its utility in predicting cognitive function and cognitive decline in later life.

An important starting point is that many scholars consider cognitive capital to be a dimension of social capital (Kim et al., 2024; Rodgers et al., 2019), even referring to it as “cognitive social capital” (Adedeji et al., 2021). Coleman (1988) developed the concept of social capital as a resource for action, recognizing that individuals act independently, albeit constrained by social norms and rules. When actors appropriate these resources, social capital can generate new cognitive capital (Nahapiet & Ghoshal, 1998), which may increase “returns to knowledge” and foster innovation (Caragliu & Nijkamp, 2014, p. 624).

We define *cognitive capital* as networks of relationships that stimulate an actor to acquire knowledge conducive to action within one’s environment and life course context, including the assessment of and response to risks and resources. Although we did not coin the term “cognitive capital,” we believe it is an underutilized concept for advancing research on the effects of neighborhoods and communities on health, especially cognitive health. Bynner and Wadsworth (2010) offered a meaningful definition of cognitive capital, but our definition is distinct in several ways. First, unlike Bynner and Wadsworth, we specify networks of interpersonal and institutional relationships as central to the structure of cognitive capital. Second, although their definition identified cognitive capital as an “accumulating asset,” we refrain from this terminology because accumulating assets typically refer to resources intended for future use rather than those that support one’s current lifestyle. Third, their definition emphasizes purposive action (leverage), whereas we view cognitive capital as manifest and potentially salubrious even if one does not intend to appropriate it. Neighborhoods with high cognitive capital may stimulate and habituate cognitive activity through peer influence and modeling.

### Conceptualizing cognitive capital

Our definition of cognitive capital has important implications for how to measure the concept, and there are different approaches to its measurement. One approach is to focus on *trust* by asking survey respondents if they trust local and central government (Adedeji et al., 2021) or believe that most people are trustworthy and willing to help people in need (Kim et al., 2024). A second approach focuses on *culture* by measuring norms, values, attitudes, beliefs, and meaning systems (Caragliu & Nijkamp, 2014; Nahapiet & Ghoshal, 1998). While we recognize that trust and a shared culture can be expressions of social capital, we believe these measures fall short of capturing the essence of cognitive capital as we define it. We summarize how cognitive capital is distinct from related concepts in Supplementary Table 1 (Pabayo et al., 2020) and outline several principles below to guide the conceptualization and measurement of it.

First, we assert that cognitive capital must be clearly linked to cognitively stimulating activities. When examining neighborhoods as ecological units of analysis, cognitive capital refers to networks of relationships within a geographic area that are likely to stimulate thinking by residents. Examples of cognitively stimulating activities include but are not limited to: reading books; visiting museums and historical sites; and appreciating art and musical performances. It should also be noted that networks engaged in stimulating cognitive activity may involve personal, family, organizational, and institutional relations.

Second, cognitive capital is propagated by resources for educational attainment, professional development, and artistic expression. Cognitive capital is structurally primed in areas that have (or are close to) institutions that promote thinking, systematic study, and higher learning. Examples include colleges, universities, fine arts schools, and local organizations that promote scientific and/or aesthetic thinking.

By corollary, the population in areas with high cognitive capital will likely manifest higher educational attainment, thereby shaping neighborhood composition. For instance, universities and schools of fine arts are likely to attract people with more years of schooling, and people with more years of schooling are likely to expect a cognitively stimulating environment replete with musical and dramatic performances as well as convocations.

Fourth, cognitive capital thrives in areas with ample health and recreational resources. Many health professionals are highly educated, and health resources provide opportunities for healthy lifestyle education, wellness checks, and therapeutics. Fitness centers and safe places to walk and/or recreate are valuable resources for maintaining physical and cognitive function.

These four elements are not exhaustive but refer to a neighborhood in which many people are learning new things, appreciating old things, and fostering a climate of thought, reflection, and well-being. Others may assume that neighborhood SES alone captures cognitive capital, but neighborhoods that stimulate learning and reflection are distinct. Socioeconomic status is related to cognitive capital, but neither variable is a simple replacement for the other. Although we articulated four elements, they are interrelated. For instance, the reciprocal relationship between institutions of higher education and a population with baccalaureate degrees illustrates how communities can build on their cognitive capital. While any one of the four elements could be used to assess cognitive capital, we specified this set to capture the core concept while also being parsimonious. Others may add to these four elements, but our point of view is that there should be a putative link to activities that stimulate cognition.

Many indicators have been tested as predictors of cognitive function in later life, including, but not limited to, crime rate, percent of households without a car, prevalence of public drunkenness, voter turnout, walkability, proximity to museums, green areas, and cafés (Besser et al., 2020; Finlay et al., 2022; Wu et al., 2020). While it is valuable that researchers have used a wide range of indicators to study how neighborhoods and local communities may be related to cognitive function in later life, the diversity of these measures can be overwhelming and raise many questions. For example, why would proximity to cafés be related to cognitive function among older adults? Cafés are an amenity, but the evidence is mixed among studies that examine their association with cognitive function or dementia (cf., Finlay et al., 2022; Wu et al., 2020).

Finally, there is a risk of a *partialling fallacy* when separately entering many indicators of neighborhood and personal characteristics into models (Gordon, 1968; Wysocki et al., 2022). Among studies that examine multiple neighborhood indicators, most treat each one as an independent variable (e.g., Choi et al., 2024; Finlay et al., 2021b), which may be fine, but ­Gordon (1968) demonstrated decades ago that the partialling fallacy is more likely to occur when the number of control variables is large and those variables are correlated.

### Conceptual development and research questions

Although there is considerable evidence that neighborhood characteristics are related to cognitive function in later life, we seek to contribute to this literature by proposing the utility of cognitive capital as an organizing concept. Others have used the term cognitive capital or proposed related terms (e.g., cognability, Finlay et al., 2022), but we are unaware of any prior study that offered a definition of cognitive capital and outlined core elements to help guide the measurement of this concept.

There has been great interest during the past decade in Stern’s (2012) specification of cognitive reserve as a resource to compensate for brain pathology in later life: lifelong “participation in cognitively stimulating activities has been suggested to slow the rate of hippocampal atrophy” (p. 1006). Moreover, he and others note that “stimulating *environments* have been associated with neurogenesis” (emphasis added; Stern, 2012, p. 1006; Clemenson et al., 2015).

Previous studies provide multiple reasons why cognitive capital, as outlined herein, may support the preservation of cognitive function in later life. First, there is substantial evidence linking better cognitive function with participation in cognitively stimulating activities among older adults, such as reading books, playing games (e.g., cards, checkers, puzzles), visiting museums, engaging in artistic expression, and writing (Iizuka et al., 2019; Wilson et al., 2002). Second, as Clarke et al. (2012) argued, neighborhood “resources have the potential to promote cognitive reserve for older adults ageing in place” (p. 736). Residing in an intellectually stimulating community aligns with the “use it or lose it” maxim to preserve function. Moreover, interactions within the neighborhood may lead directly to tangible resources while also introducing new words, ideas, and worldviews. Thus, we build on the contribution of Clarke et al. (2012) by developing the concept of cognitive capital with latent variable modeling and examining cognitive function longitudinally. Finally, there is evidence that cognitive capital is associated with better outcomes related to cognitive function, such as higher quality of life (Adedeji et al., 2021) and lower prevalence of depressive symptoms (Kim et al., 2024).

Despite these and other reasons to believe that cognitive capital is salubrious for cognitive function, we do not assume it is a panacea for cognitive decline throughout adulthood. While evidence linking cognitive capital to better cognitive function is promising, it is also possible that cognitive capital could have detrimental effects. As Wu et al. (2017) noted, “both lack of and overload of environmental stimulation may be detrimental to cognition in later life” (p. 25). Although we consider this unlikely for the elements of cognitive capital outlined herein, the detrimental thesis merits testing.

Another research question conspicuously missing from the literature concerns the age range during which cognitive capital is effective. Some exposures may be beneficial at certain ages but unhelpful for cognitive function at advanced ages (Aartsen et al., 2019). Do we observe the benefit of cognitive capital as early as age 55? And is it beneficial for people 85 or older? These questions highlight the need for further research on the temporal dynamics of cognitive capital’s influence.

We formulate three main research questions to extend our understanding of the relationship between cognitive capital and cognitive function.

1. What neighborhood indicators of cognitive capital are essential to measuring the concept?
2. Is cognitive capital prospectively related to cognitive function among people 50 years or older?
3. Is the influence of cognitive capital on cognitive function limited to select ages during later life?

## Research design and methods

### Sample

We utilize two datasets for the analysis: (1) restricted-use data from the Health and Retirement Study (HRS) and (2) the National Neighborhood Data Archive (NaNDA). The HRS is a nationally representative, biennial longitudinal survey of non-institutionalized U.S. adults aged over 50 years old, designed to explore health, economic circumstances, and aging trajectories (Sonnega et al., 2014). Although HRS data collection was launched in 1992, we specified 2010 as the baseline to match the availability of neighborhood data and because it involved a sample refresh (new cohort).

Our analysis leverages the harmonized RAND HRS data file (Bugliari et al., 2023) and the detailed HRS Cross-Wave Geographic Information Restricted Data (1992–2020). The geographic data offer detailed spatial information for census tracts and ZIP code tabulation areas (ZCTA). We used the 2010 Federal Information Processing Standards (FIPS) codes (State FIPS + County FIPS + Tract codes) to link individual-level data from HRS to NaNDA’s census tract-level variables. NaNDA provides multi-level neighborhood data encompassing physical, economic, demographic, and social factors at the census tract or ZCTA levels (Clarke et al., 2022; Finlay et al., 2020; Finlay et al., 2023; Khan et al., 2022). This linkage enables the integration of individual-level cognitive data (public availability) with contextual neighborhood characteristics (restricted availability).

The analysis uses data from 2010 to 2018, excluding 2020 to avoid potential bias due to the intrusion of the COVID-19 pandemic. Of the 22,033 participants who completed the 2010 core interview, 1,338 individuals (6.07%) were excluded for being 50 years old or younger. An additional 1,628 participants (7.39%) were excluded due to missing geographic linkage data. Furthermore, 878 participants (3.98%) were excluded for missing cognitive function data across all five waves, 20 participants (0.09%) were excluded for missing at least one control variable, and 174 participants (0.78%) were dropped due to missing sampling weights in 2010. The final analytic sample comprises 17,995 individuals, resulting in 71,399 person-wave observations.

### Measures

#### Cognitive function

Cognitive function was measured using data from the HRS Imputation of Cognitive Functioning Measures dataset (Langa et al., 2020). Respondents’ cognitive abilities were assessed through a modified version of the Telephone Interview for Cognitive Status, which includes four components: immediate recall of a 10-word list, delayed recall of the same 10-word list, serial 7s subtraction, and a backward counting task. The score for immediate and delayed recall ranges from 0 to 10, the serial 7s subtraction ranges from 0 to 5, and the backward counting task ranges from 0 to 2. These components are summed to measure cognitive function (0-27). To address missing data, HRS employed a multivariate, regression-based imputation procedure that used data from multiple waves to enhance the reliability of cognitive function estimates (Domingue et al., 2023; Langa et al., 2020, 2023).

#### Cognitive capital

We identified 28 potential indicators in NaNDA to develop a measure of neighborhood cognitive capital. These items encompassed a diverse range of neighborhood attributes, including art and educational facilities, industry, amenities, health services, SES, and demographic traits. We shared the 28 items with a panel of five gerontology scholars to identify high-priority items based on conceptual relevance and non-redundancy. After two rounds of discussion with the expert panel, they eliminated 17 of the 28 indicators (including several items that were available at the county level only). We then used confirmatory factor analysis (CFA) on the 11 items to identify a parsimonious latent construct. Based on our theoretical framework and model fit statistics, the final measurement model is based on eight indicators and accounts for one correlated error of measurement. All 28 items are listed in Supplementary Table 2, along with the three phases of measurement identification and modeling. In Supplementary Table 3, we clarified the rationale for why each indicator in Phase 2 was retained or deleted to measure cognitive capital. Additional details of the measurement modeling are described below.

#### Control variables

We included four covariates in the analysis to account for individual-level characteristics. Gender was coded as a binary variable (women = 1). Race and ethnicity were specified as five binary variables: White (reference = 0), Black, U.S.-born Hispanic, Foreign-born Hispanic, and Other. A binary variable was included to indicate whether the respondent was born in the U.S. South. We also adjusted for individual-level educational attainment because we hypothesized that living in a neighborhood with high cognitive capital may have an effect above and beyond the individual’s level of education. We coded education as an ordinal variable with four categories (< high school graduate, high school graduate, some college, and college degree or higher), focusing on degree completion (Jaeger & Page, 1996; Schafer et al., 2013). Table 1 displays descriptive statistics for the variables as well as the indicators for cognitive capital. (Due to the use of HRS restricted data, minimum and maximum values of variables are omitted.)

**Table 1.** Descriptive statistics of variables from the Health and ­Retirement Study (HRS) and the National Neighborhood Data Archive (NaNDA) (*N* = 17,995).

Characteristics	Mean/Prop.	***SD***
**Age**	66.28	10.71
**Cognitive Function (27-point TICS-m)**		
** 2010 (Wave 1)**	14.92	4.43
** 2012 (Wave 2)**	14.80	4.49
** 2014 (Wave 3)**	14.89	4.62
** 2016 (Wave 4)**	14.76	4.59
** 2018 (Wave 5)**	15.12	4.59
**Neighborhood-Level Data (Census Tracts)**		
** Museums/historical sites**	0.49	1.00
** Fine arts schools**	0.40	0.77
** Parks (open)**	2.28	2.93
** Libraries/archives**	0.43	0.78
** # Performing arts organizations**	1.33	1.97
** Fitness Centers/Gyms**	1.56	2.09
** # of Physicians**	8.50	16.59
** Proportion with bachelor’s degree**	0.25	0.17
**Cognitive Capital (8-item Latent Variable)**	0.01	0.47
**Covariates**		
** Female**	0.57	
** Born in the South**	0.33	
**Race/ethnicity**		
** White**	0.63	
** Black**	0.17	
** U.S.-born Hispanic**	0.12	
** Foreign-born Hispanic**	0.05	
** Others**	0.02	
**Educational attainment**		
** Less than high school**	0.24	
** High school**	0.29	
** Some college**	0.25	
** College and above**	0.23	

*Note*. Each covariate is measured as a binary or nominal variable. We examined the stability of census tract indicators of cognitive capital over time. Fixed effects regression revealed small annual changes in the indicators of cognitive capital. TICS = Telephone Interview for Cognitive Status.

### Analytic strategy

The analysis consisted of two main stages: (1) modeling cognitive capital as a latent variable and (2) estimating the relationships between cognitive capital and cognitive function trajectories.

After the expert panel reduced the 28 NaNDA indicators to 11, we conducted CFA within a structural equation modeling framework to compare models with varying numbers of items at two levels: census tract (*N* = 72,387) and ZCTA (*N* = 32,616). Model fit was evaluated using multiple statistics presented below. We also examined standardized factor loadings to assess the theoretical coherence of the factor structure. After finalizing the CFA model, we linked the predicted cognitive capital scores to individuals using their corresponding geocodes.

To examine whether cognitive capital was associated with cognitive function, we modeled age-related trajectories of cognitive function using multi-level (mixed-effects) growth-curve regression. We differentiated between-person and within-person effects of age by creating person-centered age (each measurement’s deviation from the individual’s own average age) and grand-mean-centered age (each person’s average age centered around the sample mean). We fitted four nested three-level models. We began this part of the analysis by estimating an unconditional baseline trajectory, established by including only within-person and between-person age terms. Because observations were nested within individuals, who themselves were nested within neighborhoods (census tracts), we allowed random intercepts at both individual and neighborhood levels, along with a random slope for within-person age at the individual level. The random intercept and random slope were allowed to correlate.

We also tested whether age had nonlinear effects on cognitive function using Wald tests and the Bayesian information criterion to evaluate the necessity of quadratic terms. These initial results indicated that a quadratic (nonlinear) effect was necessary for between-person age, which we implemented, but not for within-person age. To examine whether cognitive capital modifies cognitive trajectories, we tested interactions between cognitive capital and both within-person and between-person age. The final model added blocks of covariates—demographic, socioeconomic, and contextual factors—to assess whether the effects of cognitive capital persisted after adjusting for potential confounders.

To aid the interpretation of the relationship between cognitive capital and nonlinear cognitive function trajectory, we calculated and plotted average marginal effects (AMEs) to quantify the influence of cognitive capital on cognitive function at different ages. All analyses adjusted for sampling weights to reduce potential biases in parameter estimates and ensure the representativeness of our findings. Marginal effect tests and plots were used to provide intuitive interpretations of key relationships. Results are considered statistically significant at an alpha level of .05. All statistical analyses were completed with Stata 18.0.

## Results

### Latent variable measurement modeling of cognitive capital

**Figure 1.**
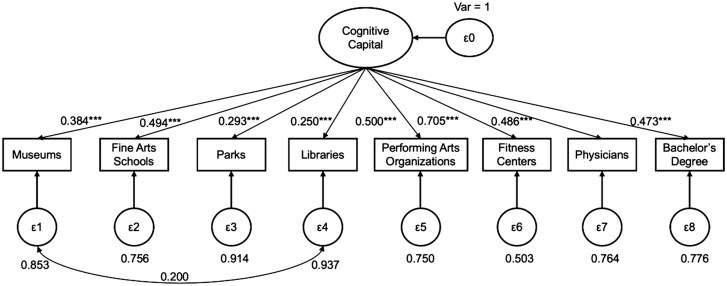
Latent variable model of cognitive capital based on U.S. census tracts (standardized solution).

We began the measurement modeling with 11 indicators at the census-tract and ZCTA levels but identified that three indicators contributed little unique information to the latent variable (amusement parks, colleges/universities, and spectator sports organizations); see Supplementary Table 2. Figure 1 summarizes the standardized factor loadings and residual variances from the final CFA model, which includes eight items measured at the census tract level. The overall model fit was good (SRMR = 0.029; RMSEA = 0.048, CFI = 0.952, TLI = 0.930; χ^2^ = 3170.1), and all factor loadings were significant (*p* < .001). Among these indicators, fitness centers showed the highest standardized factor loading (0.700), followed by performing art organizations (0.500), fine arts schools (0.494), physicians (0.486), percentage of adults with a bachelor’s degree (0.473), and museums (0.384). In contrast, libraries/archives (0.250) and parks (0.293) exhibited lower loadings, indicating weaker but still significant associations with the latent cognitive capital construct. *R*^2^ values reflected these differences, with fitness centers explaining nearly half the variance (*R*^2^ = 0.490), while libraries/archives and parks explained less than 10% each (*R*^2^ = 0.063 and 0.086, respectively). Supplementary Table 4 provides standardized results, including factor loadings, standard errors, and *R*^2^ values.

Although the factor loadings for libraries/archives and parks were lower, we retained them for two reasons. First, they were significant and, second, previous studies consider them important predictors of cognitive function (Besser et al., 2021; Song et al., 2024). Additionally, we specified one theoretically driven correlated residual between libraries/archives and museums, as both represent cultural heritage facilities that frequently co-locate.

As a robustness check, we re-estimated the measurement model with the same indicators but at the level of ZCTAs instead of census tracts (see Supplementary Table 5). Both models A and B (census-tract and ZCTA, respectively) fit the data well. The SRMR for each is below the widely accepted value of 0.08, which is indicative of a good fit (Hu & Bentler, 1999). Based on RMSEA and χ^2^, Model A fits better than Model B. Therefore, we used cognitive capital based on eight items at the census tract level in subsequent analyses.

### Cognitive capital and cognitive function trajectories

Turning to whether cognitive capital is related to cognitive function, Table 2 presents results from multi-level growth-curve models examining cognitive function trajectories. Across all models, within-person age is negatively associated with cognitive function (e.g., Model 1: β = –0.102, *p* < .001), indicating that most individuals experience cognitive decline as they age. Similarly, between-person age is consistently negative and significant (e.g., Model 1: β = –0.162, *p* < .001), showing that older individuals, on average, have lower cognitive function than their younger peers. The quadratic term for between-person age is also significant, confirming a nonlinear age trajectory. Cognitive capital shows a strong positive association with cognitive function. In Model 3, before controlling for covariates, cognitive capital significantly predicts higher cognitive scores (β  =  1.151, *p* < .001). However, in Model 4—after adjusting for demographic, socioeconomic, and contextual variables—the effect size is reduced but remains significant (β  =  0.661, *p* < .001), revealing that while some of the association is explained by covariates, cognitive capital still contributes independently to cognitive functioning. Overall, the models demonstrate both age-related decline in cognition and the potential protective role of neighborhood cognitive capital.

**Table 2.** Regression of cognitive function on cognitive capital and covariates (*N*_level-3_ = 5,780; *N*_level-2_ = 17,995; *N*_level-1_ = 71,399).

Variables	Model 1	Model 2	Model 3	Model 4
**Fixed-effects parameters**				
** Within-Person Age**	−0.102***	−0.101***	−0.101***	−0.100***
** Between-Person Age**	−0.162***	−0.139***	−0.140***	−0.126***
** Between-Person Age^2^**		−0.005***	−0.004***	−0.004***
** Cognitive Capital**			1.151***	0.661***
** BP Age × CC**			0.017	0.013
** BP Age^2^ × CC**			−0.003**	−0.003**
** Female (Ref = Male)**				0.754***
** Born in South (Ref = Born in North)**				−0.213
**Race/ethnicity (Ref = White)**				
** Black**				−0.203***
** U.S.-born Hispanic**				−1.250***
** Foreign-born Hispanic**				−0.764***
** Others**				−1.090***
**Educational attainment (Ref = Less than high school)**				
** High school**				1.805***
** Some college**				2.640***
** College and above**				3.773***
**Intercept**	14.845***	15.302***	15.227***	13.270***
**Random-effects parameters**				
**Neighborhood level (*N* = 5,780)**				
** Var (Intercept)**	11.367	11.158	10.806	7.417
	(10.917, 11.834)	(10.709, 11.626)	(10.366, 11.264)	(7.070, 7.782)
**Individual level (*N* = 17,995)**				
** Var (WP Age)**	0.034	0.034	0.034	0.034
	(0.026, 0.043)	(0.026, 0.043)	(0.026, 0.043)	(0.026, 0.043)
** Var (Intercept)**	4.894	4.780	4.773	3.753
	(4.611, 5.193)	(4.502, 5.076)	(4.494, 5.069)	(3.527, 3.993)
** Cov (WP Age, Intercept)**	0.175	0.171	0.172	0.172
	(0.131, 0.219)	(0.128, 0.214)	(0.129, 0.215)	(0.130, 0.213)
**Residuals**				
** Var (Residual)**	6.427	6.426	6.426	6.431
	(6.281, 6.576)	(6.280, 6.575)	(6.280, 6.575)	(6.286, 6.581)

*Note*. BP age refers to between-person grand-centered mean age; WP age refers to within-person age change; CC refers to cognitive capital. A product term for WP Age × CC was nonsignificant (*p *> .05). Confidence intervals for variances are in parentheses.

* *p *< .05.

** *p *< .01.

*** *p *< .001.

The random‐effects estimates indicated that neighborhoods accounted for notable variation in baseline cognition (intercept variance 11.37–7.42), while individual intercept variance also remained sizeable (4.89–3.75). Although within-person slope variance was small (0.03–0.04), the positive intercept–slope covariance (∼0.17) reveals that individuals with higher initial cognitive function experienced slower declines, consistent with widening inequalities over time, with residual variance (∼6.4) highlighting substantial within-person fluctuation left unexplained.

We tested a product term between within-person age and cognitive capital but found that it was nonsignificant, suggesting that while cognitive capital was associated with higher baseline levels of cognitive function (i.e., intercept), it did not moderate the rate of within-person cognitive change over time. In contrast, there is a significant interaction between-person age squared and cognitive capital. Given the nonlinear effect of between-person age on cognition, a helpful strategy is to visualize the relationship between cognitive capital and cognitive function by age by plotting AMEs.

**Figure 2.**
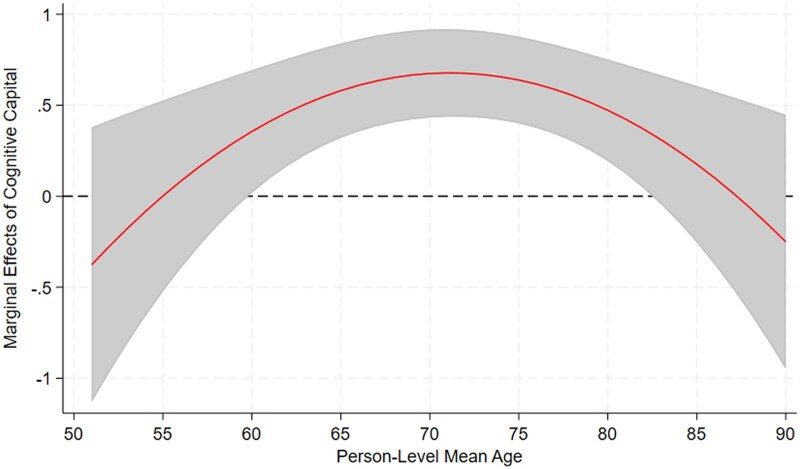
Average marginal effects of cognitive capital on cognitive function by person-level mean age (estimates in red line with 95% confidence intervals in gray shading).

As displayed in Figure 2 (developed from Model 4 of Table 2), the effect of a one-unit increase in cognitive capital on cognitive function scores varies by between-person age. A value above zero indicates a positive relationship, meaning that higher cognitive capital is associated with better cognitive function. Indeed, there are measurable benefits of cognitive capital on cognitive function during a two-decade period beginning about age 60, and the protective effect of cognitive capital is strongest at about age 70. Beyond age 82, the confidence intervals overlap with zero, indicating that the association between cognitive capital and cognitive function is no longer significant. This pattern reveals that cognitive capital plays a significant protective role from about 60 through age 82 but not thereafter.

To further examine these relationships, we conducted additional tests on AMEs to illustrate the moderating role of cognitive capital in cognitive trajectories. We estimated the AMEs of cognitive capital on cognitive function scores at four specific ages: 55, 65, 75, and 85. The results are displayed in Table 3 and quantify how much cognitive function scores are expected to change on average with a one-unit increase in cognitive capital (equivalent to one standard deviation). As shown in Table 3, cognitive capital has no meaningful effect on cognitive function at age 55 but is significantly associated with higher cognitive scores at ages 65 (d*y*/d*x* = 0.546, *p* < .001) and 75 (d*y*/d*x* = 0.639, *p* < .001). By age 85, however, the effect diminishes and is no longer statistically significant.

**Table 3.** Average marginal effects of cognitive capital on cognitive function by age (*N*_level-3_ = 5,780; *N*_level-2_ = 17,995; *N*_level-1_ = 71,399).

Age	d*y*/d*x* (cognitive capital)
**55**	0.001
**65**	0.546***
**75**	0.639***
**85**	0.176

*** *p *< .001.

### Sensitivity analysis

To check the robustness of the analysis, we conducted six sensitivity analyses by (1) excluding respondents with low cognitive scores (e.g., <7) at baseline; (2) including additional time-invariant variables (household income, depressive symptoms); (3) including several time-varying covariates (e.g., self-reported health, BMI); (4) comparing results after accounting for census-tract residential mobility (full sample *vs* stationary residents only); (5) comparing results after re-estimating Model 4 (Table 2) by adding an indicator of neighborhood SES (% of households with income >$75K); and (6) testing an interaction between individual education and cognitive capital. In each of these analyses, the beneficial association between cognitive capital and cognitive function remained.

## Discussion

Previous research has identified that some neighborhood attributes influence cognitive function in later life, but there is little consensus about which neighborhood characteristics merit study. Some studies focus on one neighborhood characteristic, such as SES (Wight et al., 2006) or proximity to parks (Besser et al., 2021), whereas others examine unique sets of neighborhood characteristics (Choi et al., 2024; Finlay et al., 2022). Each approach is useful, but few investigations sought to bridge the gap across the varied measures. Thus, we probed theoretical links between neighborhoods and human cognitive function and advanced the concept of cognitive capital. Our overarching aims were twofold.

### Conceptual development and measurement

First, we defined and measured cognitive capital for future studies of the relationship between neighborhood characteristics and cognitive function. We acknowledged the contributions of others who have used the term cognitive capital (Bynner & Wadsworth, 2010) but focused on neighborhood characteristics that are likely to stimulate cognition. There are hundreds of possible neighborhood characteristics that could be explored, but we prioritized those that are theoretically plausible and might be reasonable avenues for intervention. We could have amassed dozens of indicators but instead aimed for potential applicability across cultures and nations while also being parsimonious. We began with more than two dozen indicators but concluded that eight theoretically informed indicators captured our specification of cognitive capital.

Unlike most prior studies of the topic that entered multiple neighborhood indicators into regression models, we used latent-variable modeling to examine several goodness-of-fit tests while developing our specified construct. One advantage of this approach is its ability to reduce the number of variables by combining them into a single construct, thereby simplifying the model. Additionally, latent-variable modeling helps minimize the likelihood of biased estimates due to the partialling fallacy (Gordon, 1968; Wysocki et al., 2022). This approach integrates multiple indicators into a cohesive construct while accounting for correlated measurement errors among the indicators.

### Cognitive capital and cognitive function

We also examined whether cognitive capital was prospectively related to trajectories of cognitive function between 2010 and 2018. The trajectory analyses revealed that adults with greater cognitive capital generally demonstrated better cognitive function compared to those with lower cognitive capital. Our findings are consistent with conclusions from cross-sectional studies showing that specific indicators we used for cognitive capital—museums and proportion of residents with college degrees—were beneficial for cognitive function (e.g., Finlay et al., 2022). Similarly, some longitudinal studies have shown that living in a neighborhood with ample resources, such as public transportation, is modestly related to better cognitive function (Clarke et al., 2015). While prior studies have highlighted the benefits of selected neighborhood characteristics on cognitive function in later life, we examined the broader framework of cognitive capital.

Intrigued by the finding that high levels of environmental stimulation could be detrimental to cognitive function due to an overload of cognitive stimulation (Wu et al., 2017), we also tested for this relationship in our analysis. We found no evidence that high cognitive capital was associated with poorer cognitive function. Cognitive capital is not a panacea for cognitive decline, but the accumulated evidence is that it is either beneficial or benign.

Most prior studies adjusted for age when examining the relationship between neighborhood characteristics and cognitive functioning among older adults (Besser et al., 2021; Choi et al., 2024; Finlay et al., 2021b; Lee & Waite, 2018). Yet, we are unaware of any study that examined whether the benefits of neighborhood characteristics on cognitive function varied across the age range of respondents surveyed. Our finding that the protective effect of cognitive capital was greatest between ages 60 and 82 is novel. It also reveals that there is a roughly “60-80” window of opportunity for interventions. Cognitive capital is protective against cognitive decline in later life, but age 82 was a tipping point. This is not to imply that cognitively stimulating activities are without merit after about age 80, only that there is no evidence that cognitive capital is salubrious after age 82. To aid interpretation, we also provided a tabulation of age groups in 5-year intervals along with corresponding cognitive function scores, enabling readers to contextualize the findings across the age spectrum (see Supplementary Table 6).

There are scientific and policy implications of the 60-80 window of opportunity. First, some of the prior studies had a wide age range (≥45 years; Finlay et al., 2021b) while others surveyed a more modest range (57–85; Lee & Waite, 2018); thus, studies of various ages, including octogenarians and nonagenarians, would be informative. Indeed, we caution readers regarding the interpretation of this finding because of the relatively sparse number of HRS cases over age 85 and the possibility of survivor bias. Second, if the approximate age 80 tipping point is replicated by others, then additional resources are likely needed to preserve cognition among people 80 or older. Also, it would be helpful to investigate whether the cognitive decline at advanced ages is related to mobility limitations. Perhaps the influence of cognitive capital on cognitive function is more likely among people who are fully ambulatory to engage with neighborhood amenities.

On the methodological front, our results confirmed the utility of measuring cognitive capital with census tracts as the ecological unit, which is a commonly used approach (e.g., Clarke et al., 2015; Finlay et al., 2021b). U.S. census tracts were created to approximate neighborhoods (typically between 1,200 and 8,000 residents) and are seen as relatively small and permanent entities. Although we found that our measurement model worked well with ZCTAs, the prognostic validity of ZCTA cognitive capital on cognitive trajectories was notably weaker than models using census tracts. This is likely because ZCTAs are too coarse to capture the neighborhood heterogeneity experienced by older adults.

Consistent with prior studies, we found that the effect size of neighborhood characteristics on cognitive function is modest. Clarke et al. (2015) characterized it as “statistically significant but small” (p. 852). In our analysis, the AME of cognitive capital on cognitive function was about 0.6 for people 65 and 75 years of age after adjusting for covariates (see Table 3). To contextualize this magnitude, the effect of within-person age (aging over time) on cognitive function is roughly -0.10 per year. Thus, the effect of a one-unit increase in cognitive capital is comparable to offsetting 6 years of cognitive decline. We acknowledge that the observed AME does not meet the threshold for a conventional minimal clinically important difference but may be meaningful when viewed through a public health lens—to shift the population distribution away from impairment.

Other limitations of this study also merit attention, especially for future research. First, there is a growing literature on the long-term benefits of cognitively stimulating activities during childhood on adult cognition (Bell et al., 2025; Lewis et al., 2023). Those activities may aid brain development (Tooley et al., 2021), but we have not yet examined such a thesis. Incorporating information on early-life and midlife exposures, both positive and negative, may be helpful for advancing the literature. Although education and childhood enrichment have garnered considerable attention (Hoang et al., 2023; Lövdén et al., 2020), intellectually challenging work during middle age has a notable impact on later life cognitive function beyond the influence of education (Fisher et al., 2014; Sheftel et al., 2025). Second, we did not consider whether individuals’ physical limitations moderate the effect of cognitive capital on cognitive function, but this merits investigation. We encourage future studies to investigate these potential moderating factors to deepen our understanding of how cognitive capital shapes cognitive trajectories in later life for different sub-groups. Third, a logical next step would be to integrate the measurement of the individual’s usage of cognitively stimulating activities. Not all people who live in a neighborhood with high cognitive capital may use those resources, so it would be helpful to examine whether participation in cognitively stimulating activities boosts the influence of cognitive capital. One evidence-based intervention to consider is a campaign to foster older adults’ usage of local amenities, which could be tailored to individuals or groups. Finally, a potential limitation is that the measurement invariance of the cognitive capital construct across urban and rural census tracts has not yet been established. We speculate that the scarcity of certain amenities in rural areas may weaken factor loadings and increase standard errors, but future research should examine the construct validity of cognitive capital in diverse geographic settings.

Despite these limitations, this article contributes to the literature on later-life cognitive function in important ways. It is innovative in defining the concept of cognitive capital and identifying a set of eight indicators to measure it. The analyses also provide compelling evidence of the salubrious influence of cognitive capital on cognitive function, but the protective effect was manifested between ages 60 and 82 only. Investments to enhance cognitive capital in local communities may delay and/or reduce the likelihood of cognitive decline in later life.

## Supplementary Material

igaf115_Supplementary_Data

## Data Availability

All data files for the Health and Retirement Study used in this analysis were obtained from the HRS website: https://hrs.isr.umich.edu/data-products. Use of the restricted data was approved by the HRS Michigan Center for the Demography of Aging (MiCDA). The authors do not have permission to share the data. The study was not preregistered.
