# Supplementary material for: Does cognitive capital reduce the risk of cognitive decline in later life?
Source: Innov Aging. 2025 Oct 15;9(11):igaf115. doi: 10.1093/geroni/igaf115 (PMC12670159; doi:10.1093/geroni/igaf115)
Supplement: igaf115_Supplementary_Data [file igaf115_supplementary_data.zip › innage suppl Ferraro Han.docx]

***Innovation in Aging*** **Supplementary Material: Ferraro & Han. Does Cognitive Capital Reduce the Risk of Cognitive Decline in Later Life?**

**Supplementary Table 1.** Distinguishing Cognitive Capital from Related Concepts.

**Supplementary Table 2**. Indicators Considered for the Construct of Cognitive Capital.

**Supplementary Table 3**. Theoretical Rationale for Each Retained and Discarded Indicator Presented in Phase 2 of Supplementary Table 2.

**Supplementary Table 4**. Measurement Model Results: Standardized Factor Loadings, Standard Errors, and R² for the Cognitive Capital Construct (*N*=72,387).

**Supplementary Table 5**. Comparison of Fit Statistics for Latent Variable Measurement Modeling of Cognitive Capital with Census Tracts and ZCTA.

**Supplementary Table 6**. Cognitive Function Scores by 5-Year Age Groups (*N*=17,995).

**Supplementary Table 1.** Distinguishing Cognitive Capital from Related Concepts.

| 1. | Collective efficacy refers to perceptions of closeness (cohesion) and a generalized trust that neighbors will help others reach shared goals, but cognitive capital emphasizes *networks of relationships that stimulate knowledge acquisition*—and it is not necessarily a perception held by people in the neighborhood. |
| --- | --- |
| 2. | Cultural capital is a type of social currency based on shared values, knowledge, and behaviors that are used to operate in society and subcultures. Both cultural capital and cognitive capital emphasize knowledge accumulation, but the former prioritizes the instrumentality of knowledge to advance in social circles, whereas the latter emphasizes the sustained and intrinsic value of accumulated knowledge. |
| 3. | Neighborhood affluence refers to a favored position in a hierarchy of social status resulting from educational attainment, professional or managerial work, and high income. We anticipate that it is correlated with cognitive capital, but the focus of neighborhood affluence is material. |
| 4. | Intellectual capital is used in organizational studies and management to refer to an organization’s value (Nahapiet & Sumatra, 1998), but we specify cognitive capital as a characteristic of neighborhoods or communities. |

| **Supplementary Table 2.** Indicators Considered for the Construct of Cognitive Capital. | | | |
| --- | --- | --- | --- |
| ***28 indicators shared with expert panel^a^*** | **Phase 1** | **Phase 2** | **Phase 3** |
| 16+ civil labor force unemployed | x |  |  |
| All social services | x |  |  |
| Business/clerical schools | x |  |  |
| Elderly/disabled person services | x |  |  |
| Elementary/secondary schools | x |  |  |
| Employed civil 16+ management/professional occupations | x |  |  |
| Families with income higher than 75k | x |  |  |
| Families with incomes less than 15k | x |  |  |
| Female-headed families with kids | x |  |  |
| Home health services | x |  |  |
| Households with public assistance income | x |  |  |
| Junior college | x |  |  |
| Less than high school diploma | x |  |  |
| Nursing and residential care facilities | x |  |  |
| People with income below poverty level | x |  |  |
| Population 70+ years of age | x |  |  |
| Population non-Hispanic black | x |  |  |
| *11 indicators for measurement modeling* ^b^ |  |  |  |
| Amusement parks/arcades | x | x |  |
| Colleges, universities, & professional schools | x | x |  |
| Spectator sports organizations | x | x |  |
| Museums, historical sites, & similar institutions | x | x | x |
| Fine arts schools | x | x | x |
| Parks (open) | x | x | x |
| Libraries and archives | x | x | x |
| Performing arts organizations | x | x | x |
| Fitness centers | x | x | x |
| Physicians (number) | x | x | x |
| % population with bachelor's degree | x | x | x |

^a^ Criteria for inclusion by expert panel members: (1) Conceptual relevance*.* Retain indicators that unambiguously reflect neighborhood resources to stimulate cognition—facilities or services that promote learning, cultural engagement, physical activity, or preventative health care. Exclude items whose influence on cognition is ambiguous or that merely describe demographic composition. (2) Non‑redundancy. Prioritize parsimony (Occam’s Razor) and avoid largely duplicate measures.

^b^ Measurement modeling involved testing alternatives from 6 to 11 items before specifying an 8-item solution.

**Supplementary Table 3.** Theoretical Rationale for Each Retained and Discarded Indicator Presented in Phase 2 of Supplementary Table 2.

| **Retained items** | **Theoretical Rationale** |
| --- | --- |
| Museums, historical sites, & similar institutions. | Cognitive stimulation, especially related to history, art, science, technology, and cultural expression. |
| Fine arts schools | Cognitive stimulation related to the arts. Resources for educational attainment in creative and imaginative expression. |
| Parks (open) | Health and recreational resources for outdoor physical activity, social gatherings, and a serene environment for all ages. |
| Libraries and archives | Cognitive stimulation, especially related to reading, research, genealogy, and public lectures. |
| Performing arts organizations | Stimulates learning, reflection, and aesthetic awareness. Organizational networks to advocate for and sustain the arts via volunteer activity and/or philanthropy. |
| Fitness centers | Health and recreational resources to promote learning about physical activity, health promotion, and wellness. |
| Physicians  (number) | Health resources available locally to diagnose and treat conditions in a timely manner. Primary care and public health outreach for knowledge acquisition related to health promotion. |
| Percent population with bachelor's degree | Cognitive stimulation from neighbors and community networks to promote learning for career development and personal well-being. |
| *Items not retained* | *Theoretical Rationale* |
| Amusement parks/arcades | Although these involve recreation, activities are oriented to fun, games, and thrills. Learning may occur, but the skill may not be applicable to everyday life. |
| Spectator sports organizations | Unlike fitness centers, spectator sports involve modest physical activity, and learning may not be useful for everyday living. |
| Colleges, universities, & professional schools | Cognitive stimulation to learn new things via instruction and visiting exhibits, performances, and libraries, but likely correlated with % population with bachelor’s degree. |

Note: Items not retained manifested low standardized factor loadings in penultimate models.

| **Supplementary Table 4.**  Measurement Model Results: Standardized Factor Loadings, Standard Errors, and R^2^ Values for Cognitive Capital Construct (*N*=72,387). | | | | |
| --- | --- | --- | --- | --- |
|  | **Std. Loading** | **Std. Error** | ***p*-value** | **R^2^** |
| Museums | 0.384 | 0.004 | 0.000 | 0.148 |
| Fine arts schools | 0.494 | 0.004 | 0.000 | 0.244 |
| Parks | 0.293 | 0.004 | 0.000 | 0.086 |
| Libraries and archives | 0.250 | 0.004 | 0.000 | 0.063 |
| Performing arts organizations | 0.500 | 0.004 | 0.000 | 0.250 |
| Fitness centers | 0.700 | 0.003 | 0.000 | 0.490 |
| Physicians (#) | 0.486 | 0.004 | 0.000 | 0.236 |
| % bachelor's degree | 0.473 | 0.004 | 0.000 | 0.224 |
| Note: This table serves as a complement to Figure 1. The covariance between the errors in the total number of museums and the total number of libraries is 0.200. | | | | |

**Supplementary Table 5.** Comparison of Fit Statistics for Latent Variable Measurement Modeling of Cognitive Capital with Census Tracts and ZCTAs.

| **Fit Statistics** | **Model A** |  | **Model B** |  |
| --- | --- | --- | --- | --- |
|  | **(*N*=72,387)** |  | **(*N*=32,616)** |  |
| SRMR | 0.029 |  | 0.027 |  |
| RMSEA | 0.048 |  | 0.076 |  |
| CFI | 0.952 |  | 0.974 |  |
| TLI | 0.930 |  | 0.959 |  |
| χ^2^ | 3170.104 |  | 3365.848 |  |
| Note: Model A specifies the latent variable using eight items at the census tract level (see Figure 1). Model B includes the same eight items but at the ZIP code tabulation area level (ZCTA). SRMR = standardized root mean square residual, RMSEA = root mean square error of approximation, CFI = comparative fit index, TLI = Tucker-Lewis index, and χ^2^ = chi-square. | | | | |

**Comparison of Models**

It is reasonable to conclude that both models fit the data well. The SRMR for each model is below the widely accepted value of 0.08, which is indicative of a good fit (Hu & Bentler, 1999). Based on RMSEA and χ^2^, Model A fits better than Model B. Therefore, cognitive capital based on eight items at the census tract level is utilized in subsequent analyses.

**Supplementary Table 6**. Cognitive Function Scores by 5-Year Age Groups (*N*=17,995).

| **Age Groups** | ***N*** | **Mean** | **Standard deviation** |
| --- | --- | --- | --- |
| 51-54 | 2,829 | 15.81 | 4.01 |
| 55-59 | 3,257 | 15.74 | 4.22 |
| 60-64 | 2,544 | 15.95 | 4.27 |
| 65-69 | 2,144 | 15.58 | 4.24 |
| 70-74 | 2,685 | 14.75 | 4.29 |
| 75-79 | 1,972 | 13.82 | 4.44 |
| 80-84 | 1,248 | 12.93 | 4.14 |
| 85-89 | 703 | 11.31 | 4.25 |
| 90-94 | 261 | 10.61 | 3.78 |
| 95+ | 63 | 8.51 | 4.26 |
